# Small-Pox in Algiers

**Published:** 1865-07

**Authors:** 


SMALL-POX IN ALGIERS.—The prevalence of small-pox in Algiers has induced the French medical practitioners to instruct the natives in the practice of vaccination. It is said that this operation has now become quite popular amongst them.

